# Correlation Between Diagnosis-Related Group Weights and Nursing Time in the Cardiology Department: Cross-Sectional Study

**DOI:** 10.2196/65549

**Published:** 2025-03-04

**Authors:** Chen Lv, Yi-Hong Gong, Xiu-Hua Wang, Jun An, Qian Wang, Jing Han, Xiao-Feng Chen

**Affiliations:** 1 Department of Nursing, Beijing Chest Hospital, Capital Medical University, Beijing, China; 2 Department of Performance and Reform, Beijing Chest Hospital, Capital Medical University, Beijing, China; 3 Department of Nursing, Beijing Chest Hospital, Capital Medical University, Beijing, China; 4 Department of Information Center, Beijing Chest Hospital, Capital Medical University, Beijing, China; 5 Department of Orthopaedics, Beijing Chest Hospital, Capital Medical University, Beijing, China; 6 Department of Cardiology, Beijing Chest Hospital, Capital Medical University, Beijing, China; 7 Department of Intensive Care Unit, Beijing Chest Hospital, Capital Medical University, No.9 Beiguan Street, Area 1, Tongzhou DistrictBeijing, 101149, China, +86 18210076705

**Keywords:** diagnosis-related groups, nursing time, workload, human resources, nursing

## Abstract

**Background:**

Diagnosis-related group (DRG) payment has become the main form of medical expense settlements, and its application is becoming increasingly extensive.

**Objective:**

This study aimed to explore the correlation between DRG weights and nursing time and to develop a predictive model for nursing time in the cardiology department based on DRG weights and other factors.

**Methods:**

A convenience sampling method was used to select patients who were hospitalized in the cardiology ward of Beijing Chest Hospital between April 2023 and April 2024. Nursing time was measured by direct and indirect nursing time. To determine the distributions of nursing time based on different demographics, a Pearson correlation was used to analyze the relationship between DRG weight and nursing time, and a multiple linear regression was used to determine the influencing factors of total nursing time.

**Results:**

A total of 103 subjects were included in this study. The DRG weights were positively correlated with direct nursing time (*r*=0.480; *P*<.001), indirect nursing time (*r*=0.394; *P*<.001), and total nursing time (*r*=0.448; *P*<.001). Moreover, age was positively correlated with the 3 nursing times (direct: *r*=0.235; indirect: *r*=0.192; total: *r*=0.235; all *P*<.001). The activities of daily living (ADL) score on admission was negatively correlated with the 3 nursing times (direct: *r*=−0.316; indirect: *r*=−0.252; total: *r*=−0.301; all *P*<.001). In addition, the nursing level on the first day of admission was positively correlated with the 3 nursing times (direct: *r*=0.333; indirect: *r*=0.332; total: *r*=0.352; all *P*<.001). Furthermore, the multivariate analysis found that the nursing level on the first day of admission, complications or comorbidities, DRG weight, and ADL score on admission were the influencing factors of nursing time (*R*^2^=0.328; *F*_5,97_=69.58; *P*<.001).

**Conclusions:**

DRG weight showed a strong correlation with nursing time and could be used to predict nursing time, which may assist in nursing resource allocation in cardiology departments.

## Introduction

Measuring nursing time is beneficial to effectively avoid excess nursing labor costs from unreasonable time allocations. Nursing time is closely associated with the disease severity of patients [1], which is currently assessed through quantitative scoring systems, such as the Acute Physiology and Chronic Health Evaluation II scores and the Nursing Activity Scale [2]. However, these assessment methods are only suitable for assessing critically ill patients and have limitations [3]. The Patient Classification System (PCS) involves activities such as categorizing and quantifying the nursing level required by a patient at a given time, assigning work using scales, and calculating personnel requirements [4]. The PCS-based scientific management of nursing personnel is of great significance [5]. The diagnosis-related group (DRG) system is a diagnosis and treatment classification system that can effectively evaluate the severity of a disease classification and can combine cases based on the discharge diagnosis and careful consideration of complex disease-related factors, such as patient complications, different treatment methods, and other patient-specific differences [6]. The basic principles of DRGs are as follows: they are patient-centered; grouping is performed according to factors such as disease diagnosis, treatment methods and individual characteristics; prepaid systems are used to control costs; they encourage hospitals to improve service efficiency and quality; and they ensure reasonable costs through supervision. At the same time, grouping and payment standards are continuously optimized with the development of medical technologies to improve the quality of medical services and patient satisfaction.

DRG classification is globally recognized as an effective tool for hospital management and health care quality evaluation, and there has been research on the application of DRG systems in China since the 1980s. The DRG system divides patients into different diagnostic groups for management according to factors such as disease diagnosis, treatment methods, age, comorbidities, complications, disease severity, and disease progression [7]. In 1983, Medicare (a US federal health care insurance program) used DRG systems for the first time as a health care payment method amid health care reform. The DRG system’s prepaid model has highlighted certain advantages for controlling unreasonable medical expenses, standardizing diagnosis and treatment behaviors, enhancing patients’ experience with medical treatment, and incentivizing hospitals to strengthen their internal management. Subsequently, it has been rapidly popularized, applied worldwide, and has become one of the most dominant medical payment methods for hospitals internationally [8]. Additionally, some research has been conducted internationally on the effects of the DRG payment model on nursing, mainly including the quality of nursing [9], career development of nurses [9], nursing costs [10], and nursing time. In the meantime, studies in China have also shown that DRG systems can be used as an evaluation index for the quality of medical services, work efficiency, operating costs, and performance [11]. In recent years, a meta-analysis showed that the DRG payment model can significantly reduce the length of hospital stays and medical costs for patients, help to comprehensively and effectively control medical costs, improve the quality of medical services, and help incentivize hospitals to improve their operation and management. Furthermore, it effectively reduces the management and medical expenditures of the medical insurance department, which facilitates the formation of a standardized model for medical resource expenditures [12]. Studying the relationship between DRG weight and nursing time is important for optimizing the allocation of nursing resources, improving the quality of nursing services, promoting the implementation of the DRG payment system, and promoting the development of the nursing discipline. As an advanced medical classification and payment system, DRGs have been used worldwide, but nursing management still needs to progress simultaneously. Through in-depth research, we can accurately predict the demand for nursing resources for different DRGs, avoid the wasting of resources, and improve nursing efficiency. At the same time, GRCs help nursing staff to better identify patient needs, provide personalized services, ensure patient safety, reduce adverse events, and provide a strong support for DRG payment system reform and nursing discipline innovation.

In August 2011, the General Office of the Ministry of Health of China issued the Notice on Promoting the Application of Diagnosis-Related Groups for Hospital Evaluation, which began using DRGs for related evaluations, such as hospital service performance [13]. In 2016, the National Health and Family Planning Commission stated that the DRG system is one of the critical tools for medical quality management [14]. In 2017, the Opinions of the General Office of the State Council on Further Deepening the Guidance Reform for the Payment of Basic Medical Insurance pointed out that it is necessary to carry out pilot programs for DRGs, actively explore the framework of DRG-based payment, and promote the reform of medical insurance payment mode in China. Since nursing is a part of medical treatment, the application and implementation of the DRG system not only promotes the reform of medical treatments but also exhibits a great impact on the profession of nursing. Therefore, this study explores the correlation between DRG weight and nursing time and establishes a nursing time prediction model based on DRG weight and other factors. The model can make up for DRG-related gaps in nursing time evaluation and provide a reference for the application of DRG weight and related indicators in nursing performance appraisal and staffing.

## Methods

### Ethical Considerations

This study was conducted in accordance with the Declaration of Helsinki and approved by the Ethics Committee of Beijing Chest Hospital, Capital Medical University (LW-2024-032). This study obtained the informed consent of the patient or their family member and mainly carried out observational research and data analysis after obtaining patients' informed consent, which clearly indicated that this study will not have any negative impact on the rights and interests of the subjects and informed the patients that they do not need to give additional consent for secondary analysis of the existing data in the study. The research project adopts a method for data analysis that did not involve individual identification. For patients, this study only analyzed the costs incurred during the hospitalization of patients, without any additional time commitments of the patients. Thus, financial compensation was not given to patients.

### General Data

This was a cross-sectional study on the relationship between DRG weight and nursing time. The convenience sampling method was used to select study participants who were hospitalized in the cardiology ward of Beijing Chest Hospital between April 2023 and April 2024. The inclusion criteria were as follows: (1) patients diagnosed with angina pectoris, hypertension, sudden death, arrhythmia, heart failure, premature beat, myocardial infarction, cardiomyopathy, myocarditis, or other cardiovascular diseases and (2) patients who purchased employee medical insurance, urban and rural residents medical insurance, or commercial insurance. The exclusion criteria were as follows: (1) patients with aortic surgery, heart valve surgery, coronary artery surgery, acute myocardial infarction, major organ transplantation, haematopoietic stem cell transplantation, or severe primary pulmonary hypertension and (2) patients with multiple organ failure.

A total of 103 patients who were hospitalized aged 15‐89 (mean 58.85, SD 15.17) years were included in this study, including 67 women with a mean age of 66.2 years and 36 men with a mean age of 58.1 years. The disease diagnoses were as follows: coronary atherosclerotic heart disease (n=28), paroxysmal supraventricular tachycardia (n=1), atrial fibrillation (n=5), unstable angina pectoris (n=44), and other cardiovascular diseases (n=25). These diseases were diagnosed by coronary angiography (n=74) or interventional cardiology procedures (n=29). Patients were assessed for clear consciousness (n=102) or somnolence (n=1). Furthermore, the activities of daily living (ADL) scores on admission were between 20-100 (mean 87.41, SD 18.61) points, including scores of 100 points (n=37), 41‐99 points (n=51), and <40 points (n=15). Some patients had complications or comorbidities (n=77), including hypertension (n=47), diabetes (n=10), and others (n=30). The research was carried out in strict compliance with the Strengthening the Reporting of Observational Studies in Epidemiology (STROBE) cross-sectional checklist [15].

### Workload Measurements

#### Identification of Nursing Items

The in-hospital nursing program for patients consisted of 2 parts: direct nursing and indirect nursing. Following the Technical Operating Procedures and Quality Management Standards for Nursing [16] and the Guidelines for Clinical Nursing Practice [17], nursing staff (1 nurse, 2 senior nurses, 4 supervisor nurses and 1 chief superintendent nurse) with different nursing experiences and professional titles in the cardiology department were organized by the research team to participate in the discussion of nursing items 3 times. Ultimately, 10 direct and 12 indirect nursing items were included for the calculation (Multimedia Appendix 1).

#### Preparation

First was the selection of observers. Nurses in the department with less than 3 years of service were selected as observers and received training over the course of 3 sessions in the following: use of timers, operation timing, precautions for frequency records of direct nursing operations, requirements for indirect nursing timing, and other techniques. Digital timers were purchased and calibrated by the logistics department. The Frequency Statistics Sheet for Cardiology Patients Receiving Direct Nursing was formulated, which included the date and direct nursing items and their frequency. The statistics sheet was distributed to the nursing group of the cardiology department, and nurses were informed of the requirements for completion.

#### Calculation of Nursing Hours

After training, the observers selected 6 members of the nursing staff (2 nurses, 2 senior nurses and 2 supervisor nurses) from the cardiology department by lottery to complete each direct nursing operation. The observers calculated the average duration for each direct nursing operation from the time the nurse prepared the supplies until the completion of the operation, rounding down to the nearest second with a timer. Each nursing operation was timed by the same observer. Finally, the average duration for each direct nursing operation was calculated. Meanwhile, the nurse responsible for the patient’s nursing recorded the frequency of the direct nursing items during the hospitalization using the Frequency Statistics Sheet for Cardiology Patients Receiving Direct Nursing. Specifically, direct nursing time during hospitalization for each patient was calculated (direct nursing time = ∑average time consumption per direct nursing item × frequency of that item during hospitalization). Moreover, the indirect nursing time for patients during hospitalization was collected based on the observations from each nurse. The total nursing time per patient was equal to the sum of the direct nursing time and indirect nursing time during hospitalization.

#### Acquisition of Patients’ DRG Weights

The DRG weight refers to the average cost or total cost of a DRG case divided by the average cost or total cost of all cases in the region, respectively [18], with other external influencing factors also taken into account according to regional differences [5]. In addition to the cost of the diagnosis and treatment of diseases, calculations for DRG weights also account for the proportion of first-level nursing, the average length of hospital stays, and mortality. To a certain extent, DRG weights reflect the consumption of nursing resources. In this study, case statisticians uploaded the data to the inpatient medical service performance evaluation platform after a quality audit of the data on the first page of the discharge paperwork. Then, they obtained the corresponding DRG weights through the DRG grouping device on the platform, which were then divided into DRG weights of ≤1, >1 to ≤2, and >2, in unit intervals. The number of patients with different DRG weights reflected the distribution of disease severity.

#### Activities of Daily Living Scale

The ADL scale included 10 items of examination, with each item having a total scoring criteria between 0 and 15 points, and the maximum total score was 100 points [19]. An ADL score of 0 points indicated that the patient was completely dependent on others for daily living, while a score of 100 indicated that the patient was completely self-sufficient in their daily living. Patients with a score of <40 points had a severe impairment in their ADL, while those with 41‐60 points had a moderate impairment in their ADL, and those with >61 points had a mild impairment in their ADL. For each patient, the first assessment was performed 2 days after the stability of their vital signs or when they entered the study, and the second assessment was performed 6 months into the course of the disease.

### Statistical Analysis

The SPSS version 26.0 software package (IBM Corp) was used for the statistical analysis, and the normality test was conducted using the Kolmogorov-Smirnov method. Measurement data that were normal were expressed as mean and SD, and a 2-tailed sample *t* test was used to compare the nursing times based on the patient’s sex, complications, angiography, and surgery. The nursing time, nursing level, and DRG weight on the first day of admission were compared using a 1-way ANOVA. The Student-Newman-Keuls method was used for pairwise comparisons. Count data were expressed as frequency or rate, and the *χ*^*2*^ test was used for intergroup comparisons. Pearson correlation analysis or Spearman rank correlation analysis was used for the correlation analyses, and the multivariate analysis was performed through multiple linear regression. A bilateral *P* value of <.05 was considered statistically significant.

## Results

### Nursing Time and DRG Weights for Included Patients

**Figure 1.**
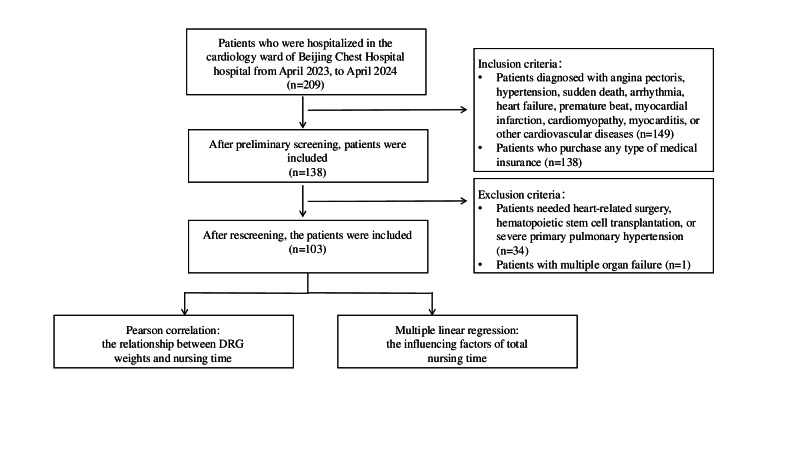
Research screening process. DRG: diagnosis-related group.

The screening process of the research is shown in Figure 1. A total of 103 subjects were included in this study. During hospitalization, the direct nursing time for patients was between 70.65‐1228.71 minutes, with a median of 335.33 (IQR 277.44-452.09) minutes. The indirect nursing time was between 30.86‐1024.30 minutes, with a median of 362.46 (IQR 229.97-584.15) minutes, and the total nursing time was between 116.48‐2169.05 minutes, with a median of 702.76 (IQR 518.55-1019.64) minutes. The average daily nursing time per patient was 134.58 minutes, and the average daily direct and indirect nursing times were 70.34 minutes and 64.24 minutes, respectively. Additionally, the DRG weights for the included patients were between 0.52‐2.57 (mean 1.51, SD 0.42; Table 1).

**Table 1.** Overall nursing time.

Variables	Values	Range
Direct nursing time (min), median (IQR)	335.33 (277.44‐452.09)	70.65‐1228.71
Indirect nursing time (min), median (IQR)	362.46 (229.97‐584.15)	30.86‐1024.30
Total nursing time (min), median (IQR)	702.76 (518.55‐1019.64)	116.48‐2169.05
DRG^a^ weights, mean (SD)	1.51 (0.42)	0.52‐2.57

a DRG: diagnosis-related group.

### Correlation Analysis of Patient Nursing Time

The results suggested that DRG weights were positively correlated with direct nursing time, indirect nursing time, and total nursing time (direct: *r*=0.480; indirect: *r*=0.394; total: *r*=0.448; all *P*<.001). Furthermore, age was positively correlated with the 3 nursing times (direct: *r*=0.235; indirect: *r*=0.192; total: *r*=0.235; all *P*<.001). The ADL score on admission was negatively correlated with the 3 nursing times (direct: *r*=−0.316; indirect: *r*=−0.252; total *r*=−0.301; all *P*<.001), and nursing levels on the first day of admission were positively correlated with the 3 nursing times (direct: *r*=0.333; indirect: *r*=0.332; total *r*=0.352; all *P*<.001), as shown in Table 2 .

**Table 2.** Correlation analysis of patient nursing time.

Item	Direct nursing time		Indirect nursing time		Total nursing time	
	Correlation coefficient	*P* value	Correlation coefficient	*P* value	Correlation coefficient	*P* value
DRG^a^ weight	0.480	<.001	0.394	<.001	0.448	<.001
Age	0.235	<.001	0.192	<.001	0.235	<.001
ADL^b^ score on admission	−0.316	<.001	−0.252	<.001	−0.301	<.001
Nursing level on the first day of admission	0.333	<.001	0.332	<.001	0.352	<.001

a DRG: diagnosis-related group.

b ADL: activities of daily living.

### Univariate Analysis of Patient Nursing Time

The results showed significant differences in the logarithmic values for direct nursing time (*t*_101_=2.230; *P*=.03), indirect nursing time (*t*_101_=2.449; *P*=.02), and total nursing time (*t*_101_=2.445; *P*=.02) between patients with or without complications. There were also significant differences between patients with or without surgeries (direct: *t*_101_=5.324; indirect: *t*_101_=4.584; total: *t*_101_=5.199; all *P*<.001), patients with different nursing levels on the first day of admission (direct: *F*_2,100_=45.421; indirect: *F*_2,100_=25.435; total: *F*_2,100_=35.495; all *P*<.001), and patients with different DRG weights (direct: *F*_2,100_=32.455, indirect: *F*_2,100_=28.581, total: *F*_2,100_=29.435, all *P*<.001). However, no significant differences were observed for the natural logarithmic values for direct nursing time, indirect nursing time, or total nursing time between patients with different sexes or for patients with or without an angiography, as shown in Table 3.

**Table 3.** Comparison of nursing time of cardiology patients with different characteristics.

Item	Direct nursing time			Indirect nursing time			Total nursing time		
	Time (ln[min]), mean (SD)	*t* test or *F* test (*df*)	*P* value	Time (ln[min]), mean (SD)	*t* test or *F* test (*df*)	*P* value	Time (ln[min]), mean (SD)	*t* test or *F* test (*df*)	*P* value
Sex		0.613^a^ (101)	.06		0.735^a^ (101)	.46		0.723^a^ (101)	.43
Male (n=36)	5.83 (0.42)			5.90 (0.67)			6.62 (0.48)		
Female (n=67)	5.85 (0.41)			5.83 (0.74)			6.55 (0.57)		
Complications		2.230^a^ (101)	.03		2.449^a^ (101)	.02		2.445^a^ (101)	.02
Yes (n=26)	5.64 (0.40)			5.64 (0.61)			6.43 (0.50)		
No (n=77)	5.94 (0.43)			5.96 (0.61)			6.66 (0.52)		
Angiography		1.682^a^ (101)	.12		1.882^a^ (101)	.06		1.911^a^ (101)	.06
No (n=29)	5.91 (0.46)			5.95 (0.71)			6.64 (0.54)		
Yes (n=74)	5.84 (0.37)			5.76 (0.70)			6.55 (0.50)		
Surgery		5.324^a^ (101)	<.001		4.584^a^ (101)	<.001		5.199^a^ (101)	<.001
No (n=70)	5.63 (0.45)			5.54 (0.73)			6.31 (0.55)		
Yes (n=33)	5.95 (0.38)			6.00 (0.61)			6.70 (0.49)		
Nursing level on the first day of admission		45.421^b^ (2, 100)	<.001		25.435^b^ (2, 100)	<.001		35.495^b^ (2, 100)	<.001
Level 2 (n=61)	5.80 (0.29)			5.73 (0.55)			6.51 (0.38)		
Level 1 (n=20)	5.67 (0.42)			5.60 (0.71)			6.40 (0.53)		
Premium level (n=22)	6.30 (0.54)			6.38 (0.90)			7.16 (0.65)		
DRG^c^ weight		32.455^b^ (2, 100)	<.001		28.581^b^ (2, 100)	<.001		29.435^b^ (2, 100)	<.001
≤1 (n=47)	5.61 (0.43)			5.44 (0.73)			6.30 (0.55)		
>1 to ≤2 (n=54)	5.91 (0.35)			5.90 (0.60)			6.60 (0.45)		
>2 (n=2)	6.27 (0.40)			6.36 (0.55)			7.09 (0.41)		

a *t* test.

b *F* test.

c DRG: diagnosis-related group.

### Multivariate Analysis of Patient Nursing Time

Stepwise regression analysis was performed with the participant’s nursing time as the dependent variable and the collected information as independent variables. Categorical variables were grouped and coded, as shown in Table 4. The results showed that when 4 variables (nursing level on the first day of admission, complications or comorbidities, DRG weight, and ADL score on admission) were included and other variables were excluded, the model *R*^2^ was 0.328, indicating that these 4 variables could explain 32.8% of the factors affecting the patients’ nursing time. In addition, the *F*_5,97_ test result of 69.58 (*P*<.001) indicated that the dependent variable fit well with these 4 variables. The Debin-Watson index was 1.842, suggesting no correlation between the independent variables of this model, and the significance test for all 4 independent variables indicated that they were statistically significant in the model and should be retained. Additionally, due to a variance inflation factor value of <10 for all 4 independent variables, there was no covariance between the respective variables, and the multiple linear regression equation was as follows:


$$Y=6.423+0.482X_{1}+0.091X_{2}-0.005X_{3}+0.167X_{4}$$

According to the standardized partial regression coefficients in the model, it can be concluded that the effect of the 4 independent variables on patient nursing time was ranked as follows (from strongest to weakest): DRG weight>nursing level on the first day of admission>ADL score on admission>complications or comorbidities (Table 5).

**Table 4.** Assigned values for variables.

Item and group		Assigned value
Nursing level on the first day of admission		
Level 1 nursing		1
Level 2 nursing		2
Premium-level nursing		3
Complications or comorbidities		
Yes		1
No		2
Surgery		
Yes		1
No		2
DRG^a^ weight		Original value
ADL^b^ score on admission		Original value
Age		Original value

a DRG: diagnosis-related group.

b ADL: activities of daily living.

**Table 5.** Multiple linear regression analysis of total nursing time. *R*=0.573, *R*^2^=0.328, adjusted *R*^2^=0.289; *F*_5,97_=69.58.

Variable	Partial regression coefficient	Standardized partial regression coefficient (SE)	*t* test (*df*)	*P* value	VIF^a^
Constant	6.423	—^b^	8.395 (101)	<.001	—^b^
DRG^c^ weight	0.482	0.446 (0.104)	8.352 (101)	<.001	2.194
Nursing level on the first day of admission	0.091	0.273 (0.110)	8.332 (101)	<.001	1.422
ADL^d^ score on admission	−0.005	−0.191 (0.004)	9.535 (101)	<.001	1.310
Complications or comorbidities	0.167	0.161 (0.089)	6.582 (101)	<.001	2.140

a VIF: variance inflation factor.

b The standardized partial regression coefficient was not calculated for the constant value.

c DRG: diagnosis-related group.

d ADL: activities of daily living.

## Discussion

By analyzing the correlation between DRG weight and nursing time, this study established a nursing time prediction model based on DRG weight and other factors. The results showed that DRG weight was positively correlated with direct nursing time, indirect nursing time, and total nursing time. The influencing factors affecting the patient’s nursing time include DRG weight, the nursing level on the first day of admission, ADL score on admission, and complications or comorbidities.

Scholars in China have learned from more advanced nursing time measurement tools that are used globally, such as the Severity of Illness Index for Pediatric Patients, Comprehensive Nursing Intervention Score, and Patient Illness Severity Nursing Index, and they have conducted weighted evaluations of factors such as project difficulty and risk, without considering factors such as the complexity of disease and treatment methods for patients. Currently, the most commonly used metric for nursing time in China is the measurement of work hours [20]; however, this is time-consuming and labor-intensive with many influencing factors, making it difficult to implement in clinical practice. Kang et al [21] performed nursing time statistics based on the hospital information platform, and despite the slightly improved efficiency, the statistical workload was still a lot due to the extraction of data from multiple systems. Meanwhile, Zhang et al [22] calculated nursing time using the load weighting method. The assignment of load weights mainly depended on the subjective assessment of experts, with a large influence from human factors. In contrast, DRG weights comprehensively considered factors such as the severity and complexity of the disease, different treatment methods, individual patient differences, and discharge outcomes [23]. These included many patient factors related to nursing time, such as the disease itself and individual characteristics, which could objectively reflect the nursing time, and the acquisition of parameters was relatively simple and easy to implement.

This study found that the average daily nursing time per patient during hospitalization in the cardiology department was 134.58 minutes. This was close to the average daily nursing time per patient (113.59 minutes) in a study by Yang et al [24] but less than the average daily total nursing time (171.02 minutes) calculated by Cai et al [25] for neurosurgery patients. This discrepancy was mainly due to the different types of diseases treated in the cardiology and neurosurgery departments, where the nursing times for neurosurgical operations were greater than that for internal medicine operations. In this study, the average daily direct nursing time per patient was 70.34 minutes, which was less than the average daily direct nursing time per patient (75 minutes) measured by Wang et al [26] for neurology patients. This was mainly due to the overall self-care ability of the cardiology patients included in this study, with 35.9% (37/103) having an ADL score of 100 on admission and 49.5% (51/103) having a score between 40‐99, while neurology patients experienced more severe functional impairments and greater dependence on care, thus increasing the burden on nursing staff. Moreover, the average daily indirect nursing time per cardiology patient in this study was 64.24 minutes, which was longer than the average daily indirect nursing time per patient (45.34 minutes) measured by Zhang et al [27]. This was directly due to the different clinical support systems of the hospitals [28], in which a well-developed clinical support system helped to reduce indirect nursing time while increasing direct nursing time.

Specifically, DRG weights regard the treatment cost of the disease as the main factor while comprehensively considering factors such as primary nursing care, average length of the hospital stay, and mortality. This was followed by obtaining the DRG weight value through data modelling, with the corresponding DRG weight value for a disease type calculated through the DRG Grouper tool. In the univariate analysis, despite the correlation between complications or comorbidities, surgery, nursing level on the first day of admission, and age, the correlation between DRG weight and nursing time was more prominent and evident. This is because DRG weights have incorporated factors related to nursing labor costs, thereby making them more valuable for implementation in the management of nursing personnel. Furthermore, the results showed that DRG weights had the greatest impact on nursing time, and although evaluating nursing time with nursing levels alone is not desirable [29], nursing levels, to some extent, reflect the severity of the disease. Moreover, the time required for some direct nursing operations was different, such as bed-making and admission assessment, which corresponded to different ADL scores. The higher the ADL score was, the less the time was required for these items, indicating a negative correlation, thereby affecting the total nursing time [29]. Furthermore, the comorbidities of cardiovascular diseases were mainly hypertension and diabetes, with 45.6% (47/103) of the patients experiencing hypertension and 9.7% (10/103) experiencing diabetes, requiring an increased monitoring of blood pressure and blood glucose, respectively, which increased nursing time. Therefore, the above factors were all related to the nursing time in the multivariate regression analysis. At the same time, the prediction of workload using a multivariate regression equation with DRG weight as the main factor can be helpful for nursing managers to measure and adjust future staffing needs based on the current staffing situation in combination with other departmental needs.

DRGs have many contributions to nursing management. They promote the standardization of the nursing process, optimize the allocation of resources, improve the efficiency of nursing work, and make nursing services more efficient and stable [24]. At the same time, DRGs promote the transformation of hospital management from extensive to refined, provide data support for nursing management departments, and enable them to make more scientific, data-based decisions [21]. In addition, DRGs help to reduce nursing risks, improve patient satisfaction, and provide more personalized nursing services for patients. Through DRG systems, hospitals can more accurately meet the nursing needs of patients and ensure patient safety. Finally, the application of DRGs puts forward higher requirements for the professional skills of nursing staff, promotes the professional development of nursing staff, and promotes the innovation of nursing research. In short, the application of DRGs has brought many positive effects to nursing management, which helps improve the quality of nursing services and promotes the modernization and scientific process of the nursing speciality.

However, this study also has some limitations. First, due to the limitation of data accessibility, this study only used the data of one hospital, and the number of cases was small. This could lead to a deviation in data selection, and there may have been some bias in the extrapolation of results. Therefore, in future studies, multicenter longitudinal studies with a larger sample size can be used for further verification. In addition, due to an untimely update of hospital disease diagnosis and surgical coding, as well as operational errors by medical staff, the data quality of the first page of medical records was low, which affected the case registration rate. The training and practical operation of medical staff on DRG-related knowledge should be strengthened before future research.

In summary, DRG weight has a strong correlation with nursing time, which can be used to predict nursing time and contribute to the allocation of nursing resources in cardiology departments. This study can improve the satisfaction of patients and their families with hospital nursing work and reduce the pain of patients. In addition, this study can also make up for the DRG-related gap in the evaluation of nursing time in cardiology departments and provide a reference for the application of DRG weight and related indicators in nursing performance evaluations and staffing.

## Supplementary material

10.2196/65549 Multimedia Appendix 1 List of direct and indirect nursing items.

10.2196/65549 Checklist 1 Strengthening the Reporting of Observational Studies in Epidemiology (STROBE) Checklist.

## Abbreviations

ADL: activities of daily living
DRG: diagnosis-related group
PCS: Patient Classification System
STROBE: Strengthening the Reporting of Observational Studies in Epidemiology
